# Case Report: Sarcoidosis mimicking head and neck cancer progression

**DOI:** 10.12688/f1000research.17834.1

**Published:** 2019-02-25

**Authors:** Edgar Pratas, João Carvalho, Isabel Domingues, Sara Pinheiro, Susana Amaral, Leila Khouri, Miguel Costa, José Eufrásio, Isonda Pires, Michael Davies, Rita Garcia, Margarida Teixeira

**Affiliations:** 1 Department of Medical Oncology, Instituto Português de Oncologia Francisco Gentil, Coimbra, Portugal; 2 Department of Radiation Oncology, Instituto Português de Oncologia Francisco Gentil, Coimbra, Portugal; 3 Department of Stomatology and Maxilofacial Surgery, Instituto Português de Oncologia Francisco Gentil, Coimbra, Portugal; 4 Department of Radiology and Medical Imaging, Instituto Português de Oncologia Francisco Gentil, Coimbra, Portugal; 5 Melanoma Medical Oncology Department, The University of Texas MD Anderson Cancer Center, Houston, USA

**Keywords:** Head Neck Cancer, Sarcoidosis, Lymph Nodes

## Abstract

Several case reports have been published describing the coexistence of sarcoidosis and cancer. In the literature, simultaneous occurrence of head and neck cancer and sarcoidosis is rarely reported.

In this paper we present a case of a 42-year-old man with squamous cell carcinoma of the oral cavity, locally advanced, which after surgery and adjuvant radiotherapy developed local persistence and progression in the mediastinal lymph nodes. The patient was submitted to chemotherapy and after a complete response, new suspicious mediastinal and hilar lymph nodes appeared in the thoracic computed tomography (CT) scan and 18F-fluorodeoxyglucose positron emission tomography (FDG-PET) scan. To enroll the patient in a clinical trial, the patient underwent mediastinoscopy with mediastinal lymph node dissection. The histopathological findings were consistent with sarcoidosis and no metastatic disease was found. Since the patient had no symptoms and the levels of serum angiotensin converting enzyme were normal, no further pharmacological intervention was done. After 4 years of follow up the patient remains without evidence of cancer.

This case shows that although imagological techniques (CT and FDG-PET scan) are extensively used to assess the tumor response, false-positive cases can occur. Whenever it is possible a biopsy of the suspected metastatic site should always be performed.

## Introduction

Sarcoidosis is a systemic disease of unknown etiology that is characterized by the development of noncaseating epithelioid granulomas in various organs, mainly the lungs and lymphatic system ^1^.

Several cases have been published describing the coexistence of sarcoidosis and cancer. It has been reported at diagnosis, during treatment, and in the surveillance of cancer patients ^2, 3^. In head and neck cancer, there are few cases reporting the simultaneous occurrence of sarcoidosis ^4, 5^.

We present a case of a patient with a squamous cell carcinoma of the right alveolar ridge of the mandible, that during treatment developed mediastinal lymphadenopathies, causing a diagnostic and therapeutic dilemma between disease progression and benign lesions.

## Case report

A 42-year-old male, Caucasian, presented to his stomatologist in May 2013 with a painless lump in the right jaw with 3 months of evolution. His Eastern Cooperative Oncology Group Performance Status was 0. The patient was an active smoker (24 pack-year) and denied drinking alcohol. He didn´t have other comorbidities. Oral cavity inspection revealed an ulcero-infiltrative lesion on the gingival margin of tooth 45 with extension to the tongue. In the lymph node assessment, an enlarged right submandibular node was palpable. The remainder of the physical examination was unremarkable. He had a normal complete blood count and biochemical profile. A biopsy of the lesion was performed and revealed a squamous cell carcinoma of the oral cavity. The patient was then referred to our institution.

**Figure 1.**
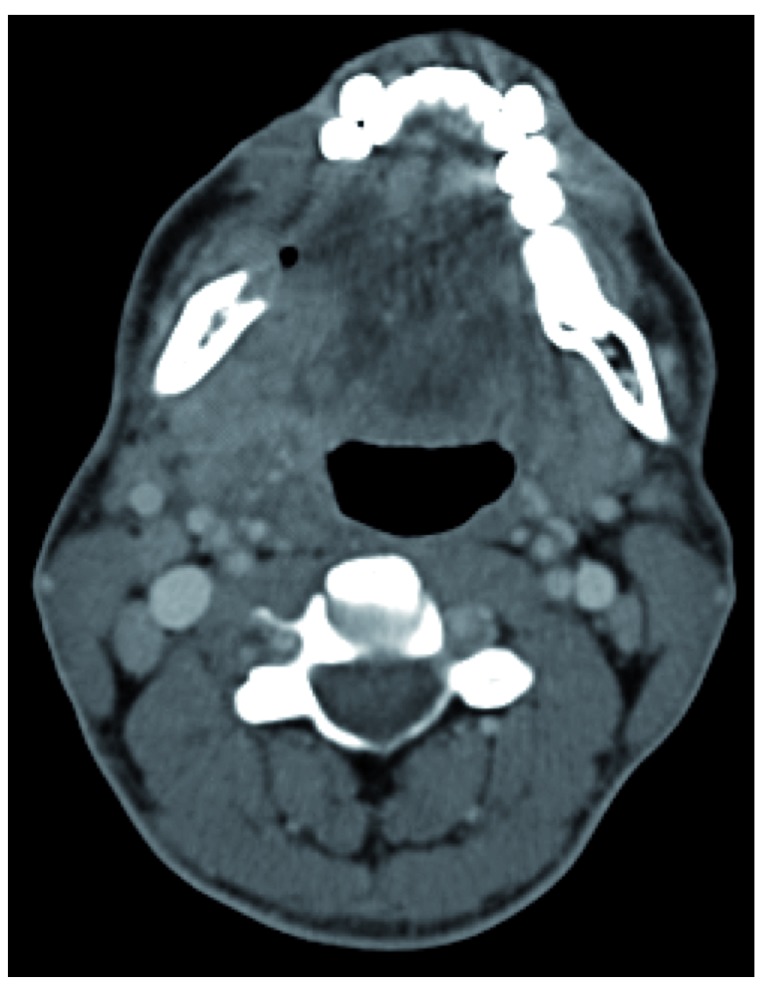
Lesion in the right alveolar ridge of the mandible with bone reabsorption; Presence of a cervical enlarged lymph nodes.

A cervical and thoracic computed tomography (CT) scan revealed a lesion in the right alveolar ridge of the mandible with bone reabsorption, and multiple bilateral enlarged cervical lymph nodes with high contrast enhancement ( Figure 1). No other anatomical changes were reported. The tumor was clinically classified in T4 N2c M0.

The case was discussed at the Head and Neck multidisciplinary team meeting (MDT) and a surgical approach was decided.

In August 2013, the patient was submitted to a partial glossectomy, right hemimandibulectomy and bilateral neck dissection. Histological analysis confirmed a squamous cell carcinoma of the oral cavity with cervical lymph node metastasis in 2 of 18 lymph nodes of the right lymph node dissection; the 12 lymph nodes dissected from the left side of the neck were clear from metastasis. The surgical margins were negative although the tumor was 1mm from the deep margin (pT4 N2b M0).

Due to the presence of positive lymph nodes, high T stage and close margins, the patient underwent adjuvant radiation therapy (60 Gy/30 fr over 6 weeks) from October to November. During the treatment he developed a grade 3 mucositis requiring a nasogastric feeding tube.

**Figure 2.**
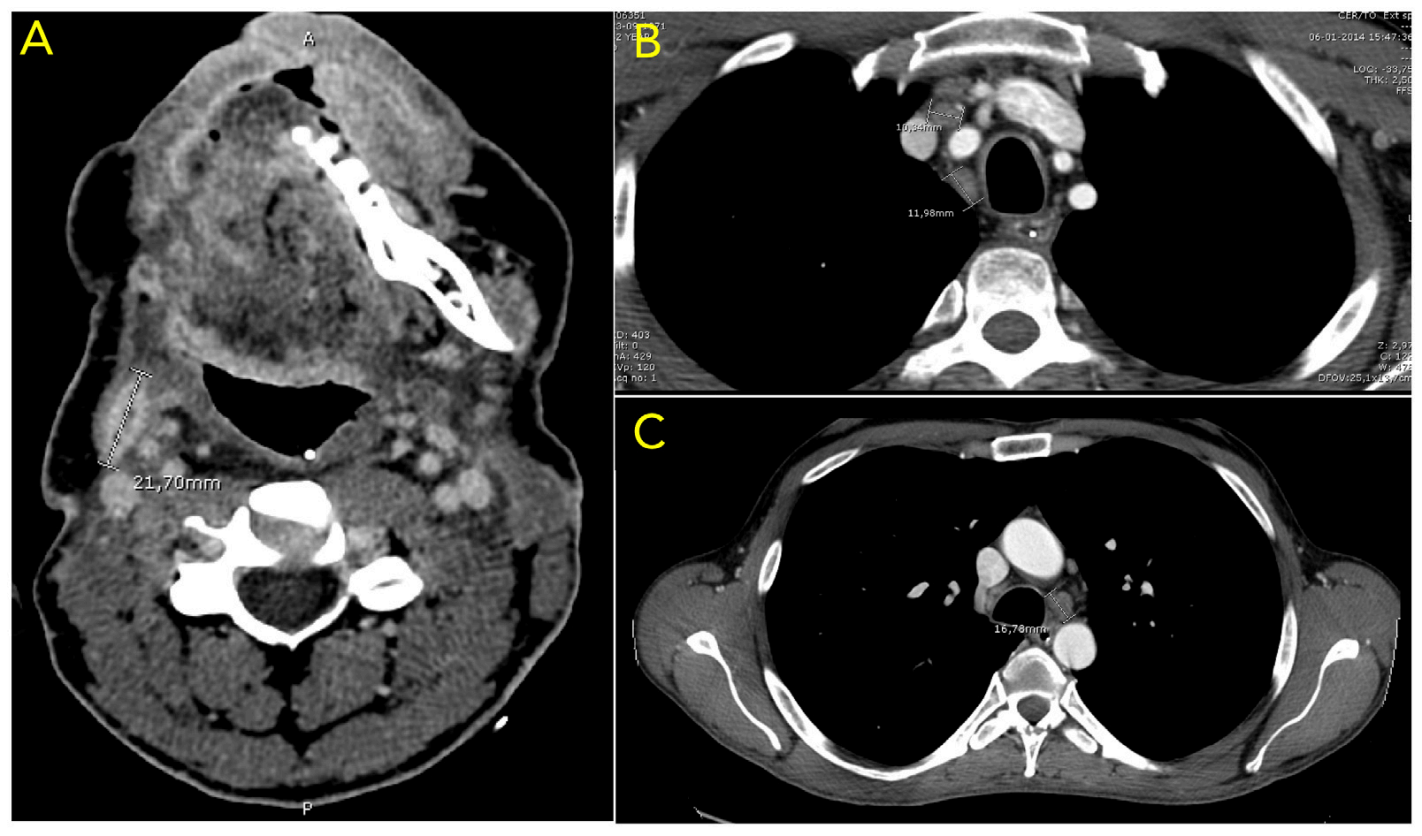
( **A**) Area of heterogenous contrast enhancement in right side of the floor of mouth and tongue as well as area of peripheral contrast enhancement with central necrosis (21,7×8 mm); ( **B**) and ( **C**) presence of enlarged mediastinal lymph nodes (16,78mm, 10,34mm and 11,98mm).

In January 2014, the cervical and thoracic CT scan revealed local and distant relapse with an area of heterogenous contrast enhancement in the right side of the floor of mouth and tongue as well as presence of mediastinal lymphadenopathies ( Figure 2). Due to this fact the patient started palliative chemotherapy with the EXTREME regimen: Cisplatin (at a dose of 100 mg/m ^2^) and cetuximab [at a dose of 400 mg/m ^2^ initially (loading dose), then 250 mg/m ^2^ on day 1 and 5-FU (at a dose of 1000 mg/m ^2^ per day for 4 days)] every three weeks. As major toxicities he developed a grade 3 skin rash and grade 2 hypomagnesemia.

After 6 cycles with clinical and imagological complete response, Cetuximab was maintained in monotherapy.

**Figure 3.**
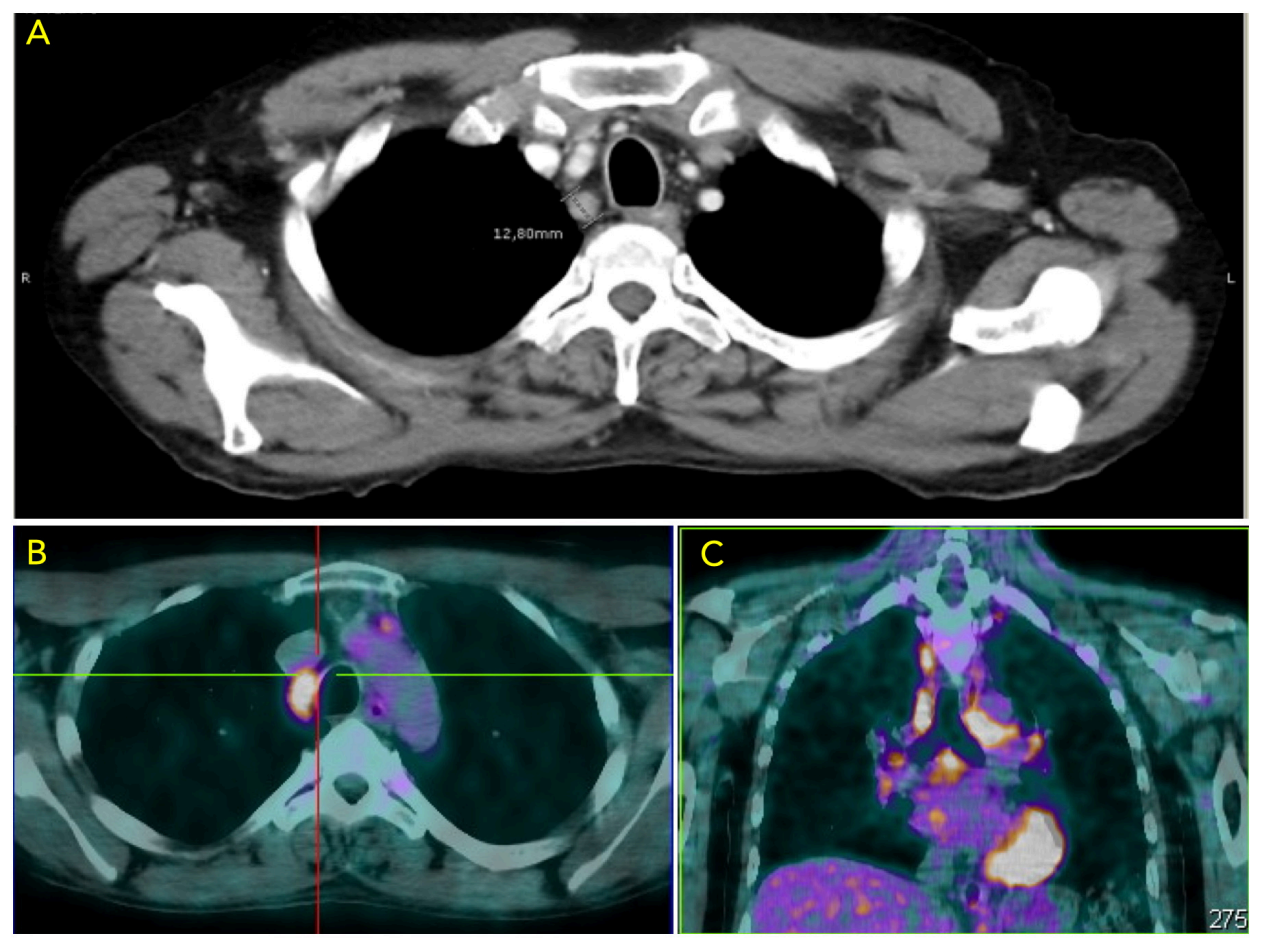
( **A**) Enlarged lymph node (12,8 mm) visible in the CT scan with correspondent abnormal in FDG uptake in the PET ( **B**); ( **C**) Focus of increased uptake in multiple mediastinal and hilar lymph nodes.

In November 2014, the control CT scan revealed tumor progression in mediastinal lymph nodes without local tumor relapse. An ^18^F-fluorodeoxyglucose positron emission tomography (FDG-PET) scan was obtained, showing hypermetabolic mediastinal and hilar lymph nodes ( Figure 3).

The Head and Neck MDT planned to enroll him in a clinical trial with immunotherapy. To meet the inclusion criteria, it was necessary to confirm histologically the mediastinal lymph node metastasis. Therefore, the patient underwent mediastinoscopy with mediastinal lymph node dissection in March 2015. Histopathological findings of the surgical specimens revealed granulomatous inflammation consistent with sarcoidosis, no metastatic disease was identified in the lymph nodes and special stains for fungi and acid-fast bacilli were negative.

The patient was referred to the Pneumology Department. Since he had no symptoms and the levels of serum angiotensin converting enzyme were normal no further pharmacological intervention was done.

In September 2017 he was submitted to a Facial Reconstructive Surgery and until now, the patient remains in follow-up without signs of local recurrence or metastasis.

## Discussion

Clinical and imagiological evaluation (CT and FDG-PET scan) are widely used to evaluate tumor response. Positive CT and FDG-PET findings in patients with suspected cancer recurrence are often used to guide therapeutic approach ^6^. However false-positives results can occur ^7^. 

We reported a rare case of a patient with a locally advanced squamous cell carcinoma of the right alveolar ridge of the mandible, that during the systemic treatment developed sarcoidosis of mediastinal lymph nodes. In our clinical case the diagnosis of sarcoidosis was incidental. The positivity in the CT scan and FDG-PET scan led us to the assumption of tumor progression. At that time, we considered the enrollment of the patient in a clinical trial because the therapeutic options were limited. The obligation of a histological confirmation of recurrent disease (inclusion criteria) led to the diagnosis of sarcoidosis and exclusion of cancer recurrence, providing a major shift in our therapeutic approach. Since then the patient remains exclusively in follow-up.

In conclusion sarcoidosis is an uncommon, but critical disease in the setting of Head and Neck cancer because it might mimic metastatic disease as the literature ^8^ and our case shows. In order to avoid incorrect treatment decisions a biopsy of the suspected metastatic site should always be performed for confirmatory diagnosis.

## Consent

Informed written consent for the publication of clinical details and images was obtained from the patient

## Data availability

All data underlying the results are available as part of the article and no additional source data are required.
