# Supplementary material for: Case Report: Sarcoidosis mimicking head and neck cancer progression
Source: F1000Res. 2019 Feb 25;8:215. [Version 1] doi: 10.12688/f1000research.17834.1 (PMC6777012; doi:10.12688/f1000research.17834.1)
Supplement: Peer review history

# Peer review: Reviewer response for version 1

**DOI:** 10.5256/f1000research.19499.r54554

**Published:** 2019-10-03

**Authors:** Christoph Spiekermann

**Affiliations:** 1 Department of Otorhinolaryngology, Head and Neck Surgery, University Hospital Münster, Münster, Germany

Pratas *et al.* describe the case of a patient with a HNSCC localized in the oral cavity. A CT scan after surgery and adjuvant radiation therapy reveals lesions with contrast enhancement in the oral cavity and mediastinal LN suspicious for tumor progression. Hence, a palliative chemotherapy was added resulting in complete response. It would be interesting to discuss whether these findings could be explained by sarcoid like lesions or sarcoidosis, as well.

 A control CT and PET-CT scan, then, revealed mediastinal LN suggestive for metastatic lesions. Biopsy of the LN leads to the diagnosis of sarcoidosis and no further therapy was conducted.

 Although histopathological findings show sarcoidosis, a residual risk of overlooking metastases in systemic inflammatory diseases still remains existent because it is not possible to take biopsies of every hypermetabolic lesion. This would be an interesting addition to the discussion section of the manuscript.

I confirm that I have read this submission and believe that I have an appropriate level of expertise to confirm that it is of an acceptable scientific standard.

# Peer review: Reviewer response for version 1

**DOI:** 10.5256/f1000research.19499.r44879

**Published:** 2019-03-04

**Authors:** Robin J.D. Prestwich

**Affiliations:** 1 Department of Clinical Oncology, St. James's University Hospital, Leeds Cancer Centre, Leeds, UK

This is a useful case report of a patient with locally advanced oral cavity carcinoma treated with surgery and adjuvant radiotherapy. CT soon after completion of treatment was suggestive of local recurrence in floor of mouth and development of new mediastinal LN. A complete radiological response was obtained following palliative chemotherapy. It would be useful to know what imaging modality this was and to explicitly state whether the local recurrence also showed a complete response.

 A subsequent PET-CT showed avid mediastinal LN strongly suggestive of distant metastases. Would be useful to comment on whether there was any uptake at primary site. Biopsy showed sarcoidosis and patient remains well several years later. It would be interesting to know the explanation for apparent local recurrence which resolved with chemotherapy - was this thought to be misinterpretation of radiology or also a sarcoid process?

 Overall a useful message regarding the importance of confirming recurrence by biopsy.

I confirm that I have read this submission and believe that I have an appropriate level of expertise to confirm that it is of an acceptable scientific standard.
